# Clinical characteristics and therapeutic strategy of frequent accelerated idioventricular rhythm

**DOI:** 10.1186/s12872-021-02221-0

**Published:** 2021-09-08

**Authors:** Lan Wang, Hailei Liu, Chao Zhu, Kai Gu, Gang Yang, Hongwu Chen, Weizhu Ju, Mingfang Li, Fengxiang Zhang, Bing Yang, Dao Wu Wang, Minglong Chen

**Affiliations:** 1 Division of Cardiology, The First Affiliated Hospital of Nanjing Medical University, 300 Guangzhou Road, Nanjing, 210029 China; 2 China State Key Laboratory of Reproductive Medicine, The Centre for Clinical Reproductive Medicine, The First Affiliated Hospital of Nanjing Medical University, 300 Guangzhou Road, Nanjing, 210029 People’s Republic of China

**Keywords:** Accelerated idioventricular rhythm, Ventricular arrhythmia, Electrophysiology, Catheter ablation

## Abstract

**Background:**

Accelerated idioventricular rhythm (AIVR) is often transient, considered benign and requires no treatment. This observational study aims to investigate the clinical manifestations, treatment, and prognosis of frequent AIVR.

**Methods:**

Twenty-seven patients (20 male; mean age 32.2 ± 17.0 years) diagnosed with frequent AIVR were enrolled in our study. Inclusion criteria were as follows: (1) at least three recordings of AIVR on 24-h Holter monitoring with an interval of over one month between each recording; and (2) resting ectopic ventricular rate between 50 to 110 bpm on ECG. Electrophysiological study (EPS) and catheter ablation were performed in patients with distinct indications.

**Results:**

All 27 patients experienced palpitation or chest discomfort, and two had syncope or presyncope on exertion. Impaired left ventricular ejection fraction (LVEF) was identified in 5 patients, and LVEF was negatively correlated with AIVR burden (*P* < 0.001). AIVR burden of over 73.8%/day could predict impaired LVEF with a sensitivity of 100% and specificity of 94.1%. Seventeen patients received EPS and ablation, five of whom had decreased LVEF. During a median follow-up of 60 (32, 84) months, LVEF of patients with impaired LV function returned to normal levels 6 months post-discharge, except one with dilated cardiomyopathy (DCM). Two patients died during follow-up. The DCM patient died due to late stage of heart failure, and another patient who refused ablation died of AIVR over-acceleration under fever.

**Conclusions:**

Frequent AIVR has unique clinical manifestations. AIVR patients with burden of over 70%, impaired LVEF, and/or symptoms of syncope or presyncope due to over-response to sympathetic tone should be considered for catheter ablation.

**Supplementary Information:**

The online version contains supplementary material available at 10.1186/s12872-021-02221-0.

## Introduction

Accelerated idioventricular rhythm (AIVR) is defined as ectopic ventricular rhythm with rate between 50 and 110 bpm at rest. It is less uncommon, but often presents as a transient, benign rhythm and results from myocardial infarction, digitalis toxicity, myocarditis, hyperkalaemia, various cardiomyopathies, and resuscitation [1–6]. A previous study suggested that no treatment is required for AIVR due to its benign clinical course [6]. However, AIVR could also be frequent, repetitive, and competitive with sinus rhythm, as observed on 24-h Holter monitoring or electrocardiogram (ECG). Such AIVR cases have unique clinical manifestations with negative impacts on patients’ prognosis. In this prospective observational study, we aimed to explore the role of catheter ablation in frequent AIVR by reviewing the related clinical characteristics/manifestations, comorbidity, treatment, and prognosis.

## Materials and methods

### Study population

From June 2002 to December 2018, 27 consecutive patients with frequent AIVR were enrolled in this study. Inclusion criteria were as follows: (1) at least three recordings of AIVR on 24-h Holter monitoring with an interval of over one month between each recording; and (2) ectopic ventricular rate at rest between 50 and 110 bpm on ECG recording. Biomarkers of myocardial injury, serum electrolytes, 24-h Holter monitoring, 12-lead ECG, and transthoracic echocardiography (TTE) were performed, and the corresponding clinical characteristics were documented. An electrophysiological study (EPS) with or without catheter ablation was performed after obtaining written consent from each participant.

### Ethics approval and consent to participate

Written informed consent to participate was obtained from all of the participants in the study and it was obtained from the parents of any participant under the age of 16. This study received a priori institutional ethics approval.

### Consent for publication

Written informed consent for publication of identifying images or other personal or clinical details was obtained from all of the participants and from the parents of any participant under the age of 18.

### Electrophysiological study and catheter ablation

EPS and catheter ablation were performed under conscious sedation. A quadripolar irrigated catheter (Navistar, Biosense Webster, Diamond Bar, CA, USA) was placed in the targeted chamber for mapping and ablation. A digital electrophysiological system was used to record intracardiac electrograms with a filtration of 30–300 Hz (WI or Bard Electrophysiology System, MA, Prucka CardioLab, General Electric Health Care System Inc., Milwaukee).

Guided by a CARTO3 or CARTO XP system (Biosense Webster Inc.), bipolar electrograms (100–150 points) were obtained during AIVR for detailed electroanatomic mapping. The origin of arrhythmia was verified by 3D mapping and/or endocardial activation recorded by a decapolar catheter along the HIS-right bundle branch (RBB) axis in case of an RBB origin [7]. At the earliest activation site, radiofrequency energy was delivered using an irrigated catheter with the maximal temperature, maximal power, and infusion rate set at 45 °C, 35 W, and 17 mL/min, respectively. Termination of AIVR during ablation together with non-inducibility and non-provocation with isoproterenol thereafter was regarded as the procedural endpoint.

### Follow-up

All patients were off anti-arrhythmic drugs (AADs) after successful ablation, except one patient (case 3) diagnosed with dilated cardiomyopathy (DCM). AADs were initiated otherwise. ECG, 24-h Holter monitoring, and TTE were performed at the 1st, 3rd, 6th, 12th month and then every year after discharge. However, ECG was performed in isolation if patients had any symptoms.

### Statistical analysis

The data were analysed by SPSS version 21.0 (SPSS Inc., Chicago, IL, USA). Continuous variables are presented as the mean ± SD if normally distributed, or as the median and interquartile range otherwise: median (X, Y). Independent samples t-test was used for comparison between groups. The Pearson correlation analysis was used to analyse the correlation between QRS width, AIVR burden, course of frequent AIVR and left ventricular ejection fraction (LVEF). Receiver operating characteristic (ROC) curve analysis was used to evaluate the diagnostic accuracy. Statistical significance was defined as a *P* value < 0.05.

## Results

### Patients characteristics

Twenty-seven patients (20 male; average age 32.2 ± 17.0 years) diagnosed with frequent symptomatic AIVR were enrolled in our study (Table 1). Two patients experienced syncope or presyncope with frequent AIVR on exertion (Case 13, Case 21). Except for one with DCM (Case 3) and another with dextrocardia (Case 20), all remaining patients were free of other comorbid cardiovascular diseases including acute myocardial infarction. Mean LVEF and left ventricular diastolic diameter (LVDd) were 57.9 ± 11.7% and 51.5 ± 10.3 mm, respectively. AIVR course duration was not correlated with LVEF (*P* = 0.402). Five patients had impaired LVEF. AADs were administered to all patients, but frequent AIVR was not terminated in any patient. Metoprolol could partially relieve the palpitations. Thyrotoxicosis, electrolyte disturbances, and acute coronary syndrome were excluded after admission.

**Table 1:** Patient baseline characteristics

Case	Gender	Symptom	Underlying disease	QRS width (ms)	LVEF (%)	AIVR burden (%)	Origin	RFCA
1	M	Palpitation	–	176	61.7	40	LV summit	+
2	M	Cough and Sputum	–	180	45.9	93.7	LV inferior wall	+
3	M	Palpitation	DCM	203	19.8	100	RV free wall	+
4	F	Palpitation	–	110	62	7.8	LAF	−
5	M	Chest discomfort	–	182	65.8	0.3	–	−
6	M	Palpitation	–	162	51	68.7	LCC	+
7	F	Palpitation	–	139	65.0	3.4	RBB	−
8	M	Palpitation	–	140	65.2	0.1	–	−
9	M	Chest discomfort	–	184	33	–	TV (6:00)	+
10	M	Palpitation	–	120	67.4	0.8	RBB	−
11	M	Palpitation	–	174	63	62.8	LCC	+
12	M	Palpitation	–	130	60	9.2	LAF	−
13	F	Syncope	–	138	68.0	66.9	RBB	−
14	M	Palpitation	–	152	–	69	LCC	+
15	M	Palpitation	–	167	69	25.3	RVOT	+
16	M	Palpitation	–	150	45.2	99.6	RBB	+
17	M	Palpitation	–	116	68.0	20.9	RBB	+
18	F	Palpitation	–	130	59	9.6	LAF	−
19	F	Palpitation	–	158	68	25.8	LCC	+
20	M	Palpitation	Dextrocardia	102	52.8	56.4	RBB	+ *
21	M	Presyncope	–	146	47.5	76.0	RBB	+
22	M	Palpitation	–	110	60	8.8	LPF	−
23	F	Palpitation	–	111	63.2	12.5	RBB	−
24	M	Palpitation	–	180	65	23.5	RV apex	+
25	M	Palpitation	–	129	59	94.6	RBB	+
26	F	Palpitation	–	160	60	45.3	LPF	+
27	M	Palpitation	–	152	61	71.5	RBB	+
Mean	–			148.2 ± 27.2	57.9 ± 11.7			

AIVR, accelerated idioventricular rhythm; DCM, dilated cardiomyopathy; F, female; LCC, left coronary cusp; LAF, left anterior fascicular; LPF, left posterior fascicular; LV, left ventricular; LVEF, left ventricular ejection fraction; M, male; RBB, right bundle branch; RFCA, radiofrequency catheter ablation; RV, right ventricular; RVOT, right ventricular outflow tract; TV, tricuspid valve

The asterisk (*) indicates AIVR failed to be ablated

### Surface 12-lead ECG and 24-h Holter monitoring features

**Fig. 1:**
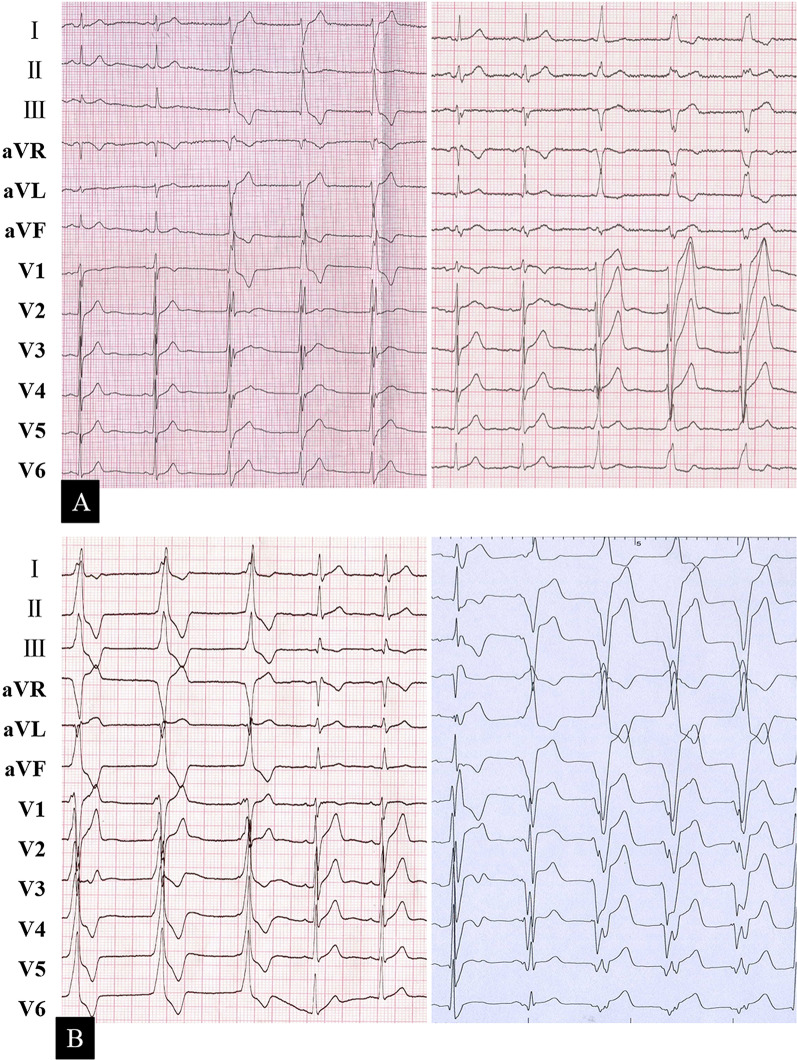
A representative ECG recording of AIVR originating from the left HPS (**a** left panel, left anterior fascicular), right HPS (**a** right panel), left ventricular working myocardium (**b** left panel), and right ventricular working myocardium (**b** right panel). Note that it is competitive with the sinus rhythm. AIVR, accelerated idioventricular rhythm; ECG, electrocardiography; HPS, His-Purkinje system

The average QRS width was 148.2 ± 27.2 ms, and it was negatively correlated with LVEF (r = − 0.43, *P* = 0.041). Typical left bundle branch block (LBBB) or right bundle branch block (RBBB) morphology indicated a His-Purkinje system (HPS) origin; otherwise, a working myocardial origin was speculated (Fig. 1). According to the morphology of the QRS complex, 15 patients had HPS focus, and the remaining had working myocardial origins. However, there was no significant difference in the LVEF (55.2% ± 16.2% vs. 59.7% ± 8.0%, *P* = 0.420) according to different AIVR origins.

**Fig. 2:**
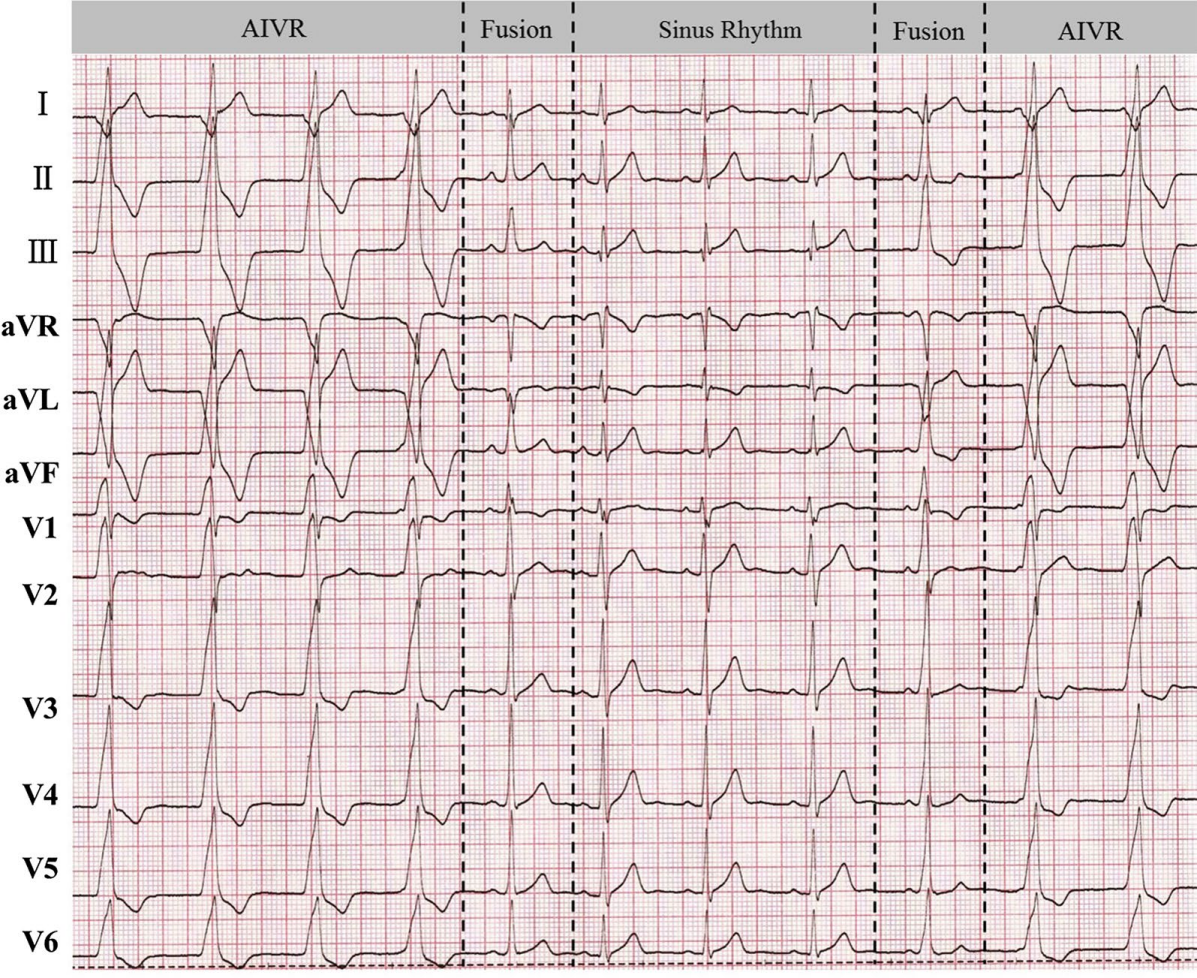
The phenomenon of AIVR competing with the sinus rhythm. AIVR, accelerated idioventricular rhythm

**Fig. 3:**
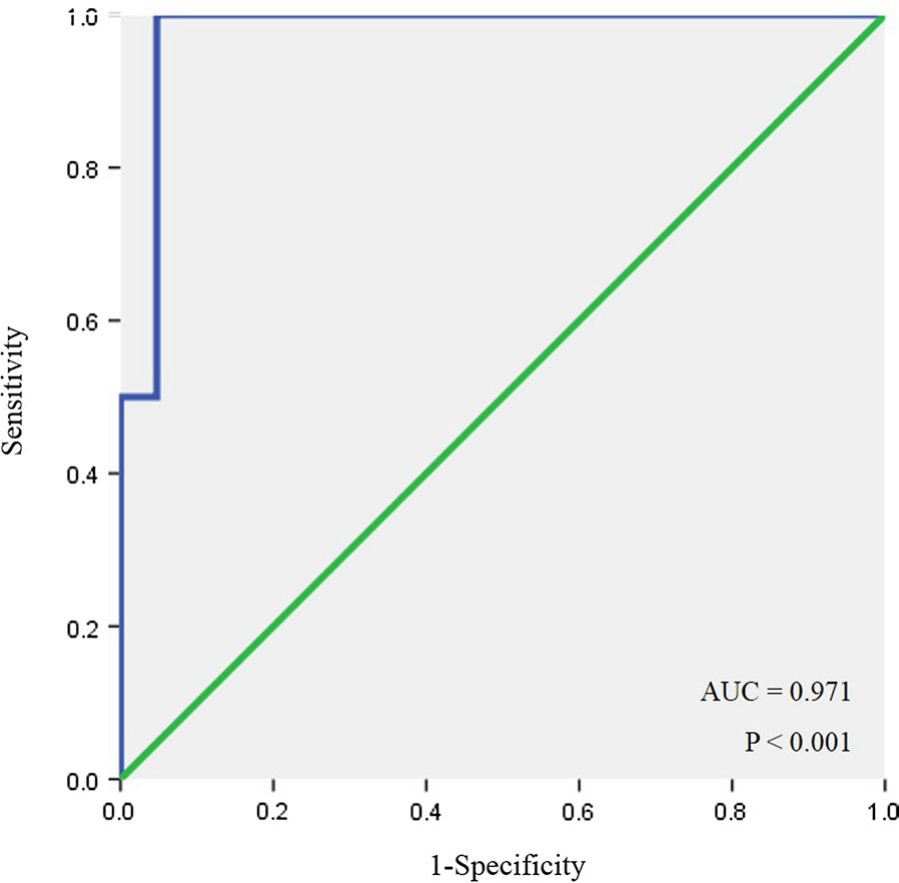
ROC curve of AIVR burden for predicting impaired LVEF. The AUC was 0.971. AIVR burden was negatively correlated with LVEF (*P* < 0.001). An AIVR burden of over 73.8%/day was predictive of impaired LVEF (< 50%), with a sensitivity of 100% and a specificity of 94.1%. AIVR, accelerated idioventricular rhythm; LVEF, left ventricular ejection fraction

AIVR competed with the sinus rhythm (Fig. 2), and its burden varied from 0.1 to 100% in patients subjected to 24-h Holter monitoring. AIVR burden was negatively correlated with LVEF (r = − 0.678, *P* < 0.001). An AIVR burden of over 73.8%/day was predictive of impaired LVEF (< 50%), with a sensitivity of 100% and a specificity of 94.1%. The AUC was 0.971 (Fig. 3).

### Electrophysiological findings and catheter ablation

**Fig. 4:**
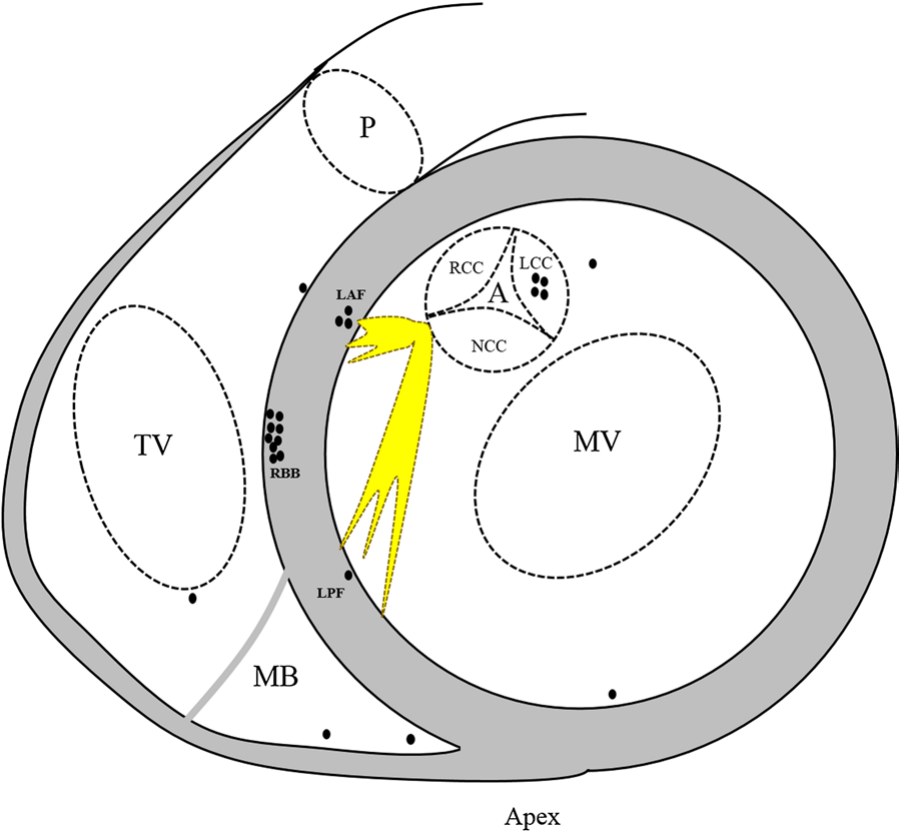
Schematic diagram of the distribution of frequent AIVR foci. A, aortic annulus; AIVR, accelerated idioventricular rhythm; LAF, left anterior fascicular; LCC, left coronary cusp; LPF, left posterior fascicular; MB, moderator band; MV, mitral valve; NCC, noncoronary cusp; P, pulmonary annulus; RBB, right bundle branch; RCC, right coronary cusp; TV, tricuspid valve

**Fig. 5:**
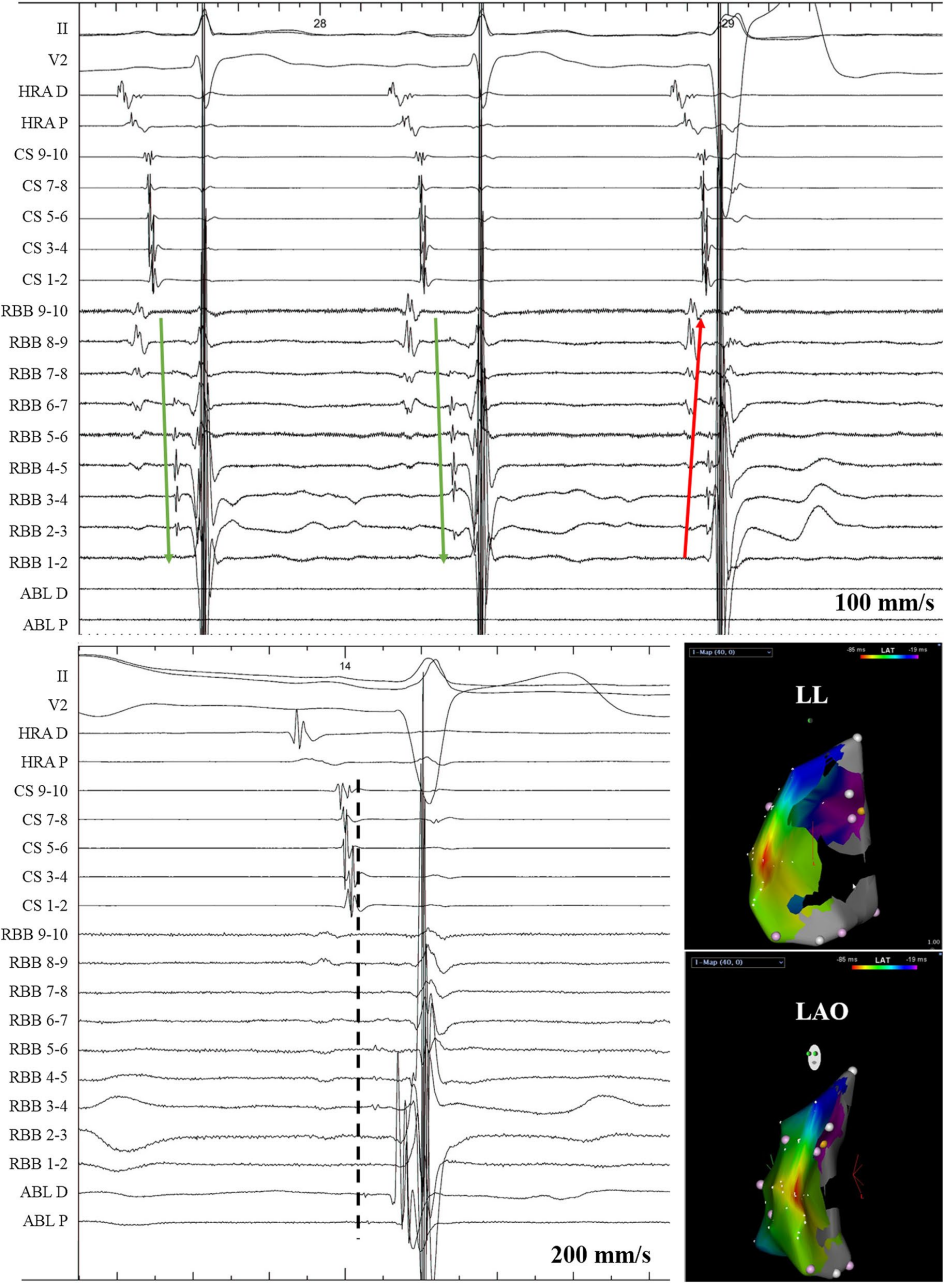
Ablation of AIVR with an RBB focus (Patient 27). A decapolar catheter was placed along the HIS-RBB axis. Upper panel: The activation of HIS-RBB went from proximal to distal (green arrow), but was reversed during AIVR (red arrow). Lower panel: The ablation target on the endocardial electrogram and three-dimensional mapping. Please note the advanced Purkinje potential on ablation catheter. ABL, ablation catheter; AIVR, accelerated idioventricular rhythm; CS, coronary sinus; HRA, high right atrium; RBB, right bundle branch

EPS and catheter ablation were recommended if the clinical status met one of the following criteria: (1) over 20% AIVR burden; (2) impaired LVEF; (3) syncope or presyncope due to over-response to sympathetic tone which was defined as sustained ventricular tachycardia (110–250 bpm) with the same QRS morphology as AIVR on exertion. Among all subjects, 18 patients met the criteria, and one (Case 13) refused interventional procedures (Table 1). The rest of the patients received ablation; foci distribution is demonstrated in Fig. 4. Case 20 failed due to dextrocardia with septal membranous aneurysm, which prevented the catheter from approaching the earliest activation site. In Case 1, the earliest target site was identified at the left ventricular summit, and radiofrequency ablation successfully suppressed AIVR, but it recurred on the second day after ablation. A second procedure was attempted with the bipolar ablation technique, and the focus was eliminated after bipolar ablation between the left coronary cusp and the corresponding spot at the left ventricular summit. Six patients with right ventricular HPS focus received ablation (Table 1) and an example of endocardial electrogram and three-dimensional mapping of patient 27 is shown in Fig. 5. Among these six patients, AIVR recurred in cases 16 and 17, and successful ablation was achieved at a more proximal level during the second procedure. Case 3 with DCM received an emergent ablation because of persistent AIVR with a precarious haemodynamic state, and the clinical outcome was improved after successful ablation on the free wall of the right ventricle.

### Follow-up

Metoprolol was prescribed to those who did not receive ablation, those with failed ablation, and the patient with DCM (Case 3). Symptoms were relieved in all subjects with successful ablation and partially relieved in those who received medication with a median follow-up period of 60 (32, 84) months. Although LVEF was improved from 19.8 to 30.6% in the DCM patient after ablation with symptomatic relief, he succumbed to sudden cardiac death 5 months post discharge. Patient 13, who refused ablation, died two months post discharge because of AIVR over-acceleration provoked by an unexplained infection. The LVEF of the remaining patients with impaired cardiac function who underwent ablation completely recovered 6 months after discharge. The mortality rate of this cohort was 7.41%.

## Discussion

The major findings of this study were as follows: (1) Unlike transient asymptomatic AIVR, repetitive symptomatic AIVR has its unique clinical characteristics and does have an adverse effect on prognosis; (2) Catheter ablation should be considered for AIVR patients with arrhythmia burden over 70%, impaired LVEF, and/or symptoms of syncope or presyncope due to over-response to sympathetic tone.

### Diagnosis, clinical manifestations, and management

Diagnosis of repetitive AIVR is mainly based on ECG and Holter monitoring. The ECG characteristics are identical to those of transient AIVR [8], with the exception of a higher burden recorded on Holter monitoring. To the best of our knowledge, there is no exact definition of repetitive AIVR that is of clinical significance. Therefore, screening and stratifying this patient population are of clinical importance.

Transient AIVR is mostly observed in patients during the reperfusion phase following acute myocardial infarction, with structural heart disease or predisposing factors, and is usually tolerable with no impact on prognosis. However, per our observation, repetitive AIVR seems to be different from transient AIVR in terms of clinical manifestations and prognosis. Although it most likely occurs in patients with a structurally normal heart, we did encounter a DCM patient with persistent AIVR that worsened the clinical outcome. Frequent AIVR may cause arrhythmia-induced cardiomyopathy (AIC) [9, 10]. In our patient cohort, impaired LVEF was completely reversed to normal in four patients post-ablation. This is similar to premature ventricular complexes (PVCs), which can cause AIC with a high ectopic burden [11, 12]. Although the DCM patient with heavy AIVR burden in our observational group had partial clinical improvement after successful ablation, we speculated that frequent AIVR increases the clinical risk in patients with structural heart disease and therefore worsens their clinical course. Previous studies found that duration of symptoms, absence of symptoms, and epicardial origin of PVCs were independently associated with PVC-induced cardiomyopathy [13, 14]. However, except AIVR burden and QRS width, a tentative analysis did not find a correlation between impaired LVEF and other factors such as origin and course of frequent AIVR. Unlike those with PVCs, all patients in our cohort were symptomatic, which might be attributable to the following reasons: (1) Compared with asymptomatic patients, symptomatic patients were more inclined to consult a cardiologist; (2) As AIVR is continuous for at least three beats, it is easier to experience discomfort compared with PVCs. Additionally, none of the subjects in our cohort had an epicardial origin. Hence, whether an epicardial origin could affect LV function in AIVR patients remains unknown. The cut-off value of AIVR burden yielded from the current study to predict impaired LV function seemed to be higher than that of PVCs [15, 16]. Unlike PVCs with frequent short coupling intervals, AIVR has a relatively regular RR interval, and the heart rate during AIVR is almost within normal range. This might be the potential reason for a higher AIVR burden in predicting impaired LV function than PVCs. However, additional cases should be included in future studies to increase prediction accuracy. Like frequent PVCs, frequent AIVR often begets palpitation due to its ectopic and competitive nature. Despite this, an overreaction to the sympathetic tone of AIVR could result in haemodynamic instability and even cardiogenic shock, especially in patients with structural heart disease.

**Fig. 6:**
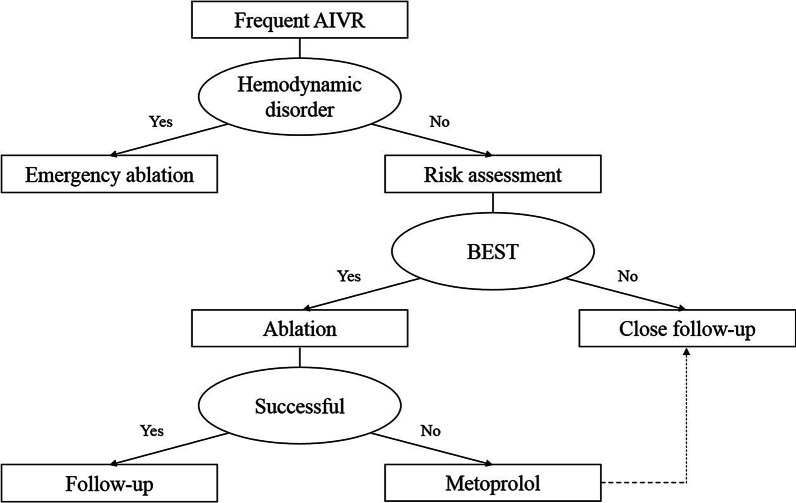
Flowchart depicting frequent AIVR management. BEST is the abbreviation of the following criteria: over 70% Burden, impaired LVEF, or Syncope or presyncope due to over-response to sympathetic Tone. AIVR, accelerated idioventricular rhythm

Unlike transient AIVR, which requires no treatment, frequent AIVR requires optimal treatment, and catheter ablation is the utter resort in some patients. Indications for catheter ablation are as follows: (1) over 70% AIVR burden; (2) impaired LVEF; and (3) syncope or presyncope due to over-response to sympathetic tone. Metoprolol could be prescribed for all other patients, and close follow-up is necessary (Fig. 6).

### Underlying mechanisms

Ectopic automaticity and autonomic imbalance seem to play important roles in the generation of transient AIVR [17]. Compared with transient AIVR, frequent AIVR has similar ECG characteristics regarding gradual warm-up and cool-down phenomena. Triggered activity [18] usually underlies idiopathic ventricular arrhythmia, manifesting a regular coupling interval in ECGs, which is different from what we have observed in the current study. Over 90% of the repetitive AIVR patients were free of any known causes in our study; hence, it remains unknown whether they had coexisting cardiomyopathy. Nevertheless, this subtype enriches the spectrum of ventricular arrhythmias.

### Study limitations

Although this study was a prospective cohort study, bias may have occurred due to the relatively small sample size. Additionally, all the patients were symptomatic as asymptomatic patients were less likely to consult a cardiologist, thereby resulting in a potential bias. Comprehensive clinical and laboratory investigations to disclose the mechanism and aetiology of frequent AIVR were not conducted. Moreover, some patients did not receive ablation and the exact foci remained unconfirmed. Thus, a larger cohort study with comprehensive enrolment is necessary.

## Conclusions

Frequent AIVR with a heavy burden has a unique clinical manifestation and prognosis regardless of its aetiology. Ablation is recommended in those with a heavy burden, impaired LVEF, or symptoms of syncope or presyncope due to over-response to sympathetic tone. Metoprolo can be used to partially relieve symptoms. Long-term follow-up should be considered in such AIVR patients.

## Supplementary Information

**Additional file 1.** Raw data of all participants.

## Data Availability

All data generated or analysed during this study are included in this published article.
