# Supplementary material for: Clinical characteristics and therapeutic strategy of frequent accelerated idioventricular rhythm
Source: BMC Cardiovasc Disord. 2021 Sep 8;21:425. doi: 10.1186/s12872-021-02221-0 (PMC8427942; doi:10.1186/s12872-021-02221-0)
Supplement: Supplementary file 1 — Additional file 1. Raw data of all participants. [file 12872_2021_2221_MOESM1_ESM.pdf]

QRS	LVEF	Burden
176.0	61.70	40.00
180.0	45.90	93.70
203.0	19.80	100.00
110.0	62.00	7.80
182.0	65.80	0.30
162.0	51.00	68.70
139.0	65.00	3.40
140.0	65.20	0.10
184.0	33.00	#NULL!
120.0	67.40	0.80
174.0	63.00	62.80
130.0	60.00	9.20
138.0	68.00	66.90
152.0	#NULL!	69.00
167.0	69.00	25.30
150.0	45.20	99.60
116.0	68.00	20.90
130.0	59.00	9.60
158.0	68.00	25.80
102.0	52.80	56.40
146.0	47.50	76.00
110.0	60.00	8.80
111.0	63.20	12.50
180.0	65.00	23.50
129.0	59.00	94.60
160.0	60.00	45.30
152.0	61.00	71.50
